# Introduction of the Buddy Scheme to First Year Core Trainees (CT1s) in the West Midlands Deanery

**DOI:** 10.1192/bjo.2022.292

**Published:** 2022-06-20

**Authors:** Amy Burlingham, Louay Eltagy, Hari Shanmugaratnam

**Affiliations:** 1 Coventry and Warwickshire Partnership NHS Trust, Coventry, United Kingdom; 2 Birmingham and Solihull Mental Health NHS Foundation Trust, Birmingham, United Kingdom; 3 Black Country Healthcare NHS Foundation Trust, Wolverhampton, United Kingdom

## Abstract

**Aims:**

Following a reflective session in the Birmingham MRCPSYCH course organized by West Midlands Deanery, CT1s identified the need for an informal peer support mechanism that bridged the gap between what is expected of them and the challenges of adjusting to the training scheme. This need became even more apparent during the COVID-19 era. This led to the creation of the buddy scheme. The main aims of the scheme are to design and develop a sustainable mechanism by which core trainees in higher years can support their year 1 counterparts informally, ease the transition of CT1 trainees into training and eliminate obstacles to success and reduce the differential attainment that hinders international medical graduates’ (IMGs) outcomes.

**Methods:**

The pilot phase started on August 2020. During this phase 3 trusts in the West Midlands were approached to share in the scheme. A fourth trust already had their own local buddy scheme. Only 1 trust shared in the process at the beginning and a second one joined later. The scheme was coordinated by the local Post Graduate Medical Education Departments in the respective trusts. All CT1s newly joining the training program were paired with a more experienced core trainee (CT2) who had their respective job the previous year. Next phase started in August 2021. During this phase all 3 trusts shared in the scheme from the beginning. A training session on the expectation from CT2s was conducted for them. A higher trainee was allocated to coordinate the process for each trust. CT2s were advised to meet their buddies at least once a month in the first 3 months.

**Results:**

A total of 24 CT1s shared in the pilot phase. All of them found the training either good or very good. 57% of CT1s found the scheme helpful in easing the transition into training and made them more confident in fulfilling their role. Most of them communicated with their buddies 1–2 times in the pilot phase. In the second phase around 40 CT1s shared in the scheme. Around 80% of CT1s found the scheme helpful and recommended that it continues. There was more contact between buddies at this stage.

**Conclusion:**

All trainees found it easy to approach their buddy and would consider becoming a buddy next year. The most discussed topics were portfolio, work-place based assessments, expectations of the day job and on-call duties, followed by exams and end of year assessments (ARCP).

